# Case Report: Multiple autoimmune syndrome (MAS)—An unusual combination

**DOI:** 10.3389/fped.2022.1054025

**Published:** 2022-11-14

**Authors:** Elaine Yi Lee Kwong, Manson Chon In Kuok, Winnie Kwai-Yu Chan

**Affiliations:** Department of Pediatrics, Queen Elizabeth Hospital, Hong Kong, Hong Kong SAR, China

**Keywords:** multiple autoimmune syndrome (MAS), diabetes mellites (DM), autoimmune thyroid desease, systemic lupus erythematosus, kikuchi - fujimoto disease

## Abstract

This is a case report of a Chinese adolescent boy who had multiple autoimmune syndrome (MAS) of a rare combination comprising type 1 diabetes, Hashimoto thyroiditis and childhood-onset systemic lupus erythematosus (SLE). He developed SLE within one year of symptom onset, presenting with necrotising histiocytic lymphadenitis and hepatitis. We highlight the importance for physicians to be aware of the need for continued surveillance for developing new autoimmune diseases in patients with multiple autoimmune diseases. It is also essential to remain vigilant for overlap syndrome as autoimmune diseases commonly share similar subphenotypes and non-specific autoantibodies. Long-term follow-up is warranted to define the final phenotype.

## Case history

A 12-year-old Chinese adolescent boy presented with a two-week history of polydipsia, polyuria, nocturnal enuresis and weight loss. His blood tests showed high glucose (26.7 mmol/L), beta-hydroxybutyrate (3.59 mmol/L), metabolic acidosis and presence of anti-islet cells. He was diagnosed with type 1 diabetes with ketoacidosis. He was also incidentally found to have Hashimoto thyroiditis with the presence of anti-thyroglobulin and anti-thyroid peroxidase antibodies.

One year later, he presented with a prolonged remittent and intermittent fever of more than three weeks. He had a painless swelling on the right side of his neck which progressively increased in size. He had no constitutional symptoms. He had no family history of autoimmune diseases. Upon physical examination, a chain of enlarged lymph nodes measuring 2.5 cm was noted in the right upper cervical region. The nodes were firm and non-tender without overlying skin changes. Enlarged lymph nodes were also present in the left cervical region but to a lesser extent. Shotty lymph nodes were noted in the groins bilaterally. The rest of the systemic examination was normal.

His blood tests showed microcytic anaemia (hemoglobin 9.6 g/dl) due to iron deficiency, leukopenia (3.4 × 10^9^/L), lymphopenia (0.8 × 10^9^/L) and normal platelet count of 202 × 10^9^/L (Table 1). The reticulocyte was 0.4%. The peripheral blood smear showed hypochromia and microcytosis without abnormal blasts. The clotting profile was normal. Inflammatory markers were elevated. C-reactive protein (CRP) was 17 mg/L (reference <5 mg/L). Erythrocyte sedimentation ratio (ESR) was 76 mm/h (reference <17 mm/h). Lactate dehydrogenase (LDH) was 358 IU/L (reference interval 118–221 IU/L). Ferritin was 1,166 pmol/L (reference interval 32–342 pmol/L). Triglyceride was normal at 1.3 mmol/L (reference <1.7 mmol/L). There was a reversal of the albumin to globulin ratio. Albumin was 32 g/L (reference interval 37–47 g/L). Globulin was 47 g/L (reference range 24–37 g/L). He had normal serum bilirubin. Liver enzymes were normal initially but started to rise on day 8 of admission. They peaked on day 12 of admission with aspartate transaminase (AST), alanine aminotransferase (ALT), gamma-glutamyl transferase (GGT) and alkaline phosphatase (ALP) at 424 IU/L (reference <42 IU/L), 307 IU/L (reference <51 IU/L), 105 IU/L (reference <42 IU/L) and 272 IU/L (reference interval 74–290 IU/L) respectively, suggestive of predominantly hepatocellular pattern of liver injury. Viral markers including hepatitis A, B and C, Epstein-Barr virus (EBV), cytomegalovirus (CMV), human immunodeficiency virus (HIV), dengue virus, Japanese E encephalitis virus (JEV) were negative. Bacterial cultures from peripheral blood, bone marrow, urine, stool and throat yielded no growth. Serologies for atypical organisms were negative, including Salmonella typhi, Bartonella henselae, Brucella abortus and melitensis, and Toxoplasma gondii. Antinuclear antibodies (ANA) were present at 1:640 with a speckled and cytoplasmic pattern. Anti-double-stranded DNA (dsDNA) was mildly elevated at 69 IU/ml (reference ≤50 IU/ml). The screening of autoantibodies against extractable nuclear antigens (ENA) showed the presence of anti-ribonucleoprotein/Smith (RNP/Sm). Anti-Smith (Sm), anti-Ro, anti-La, anti-Scl-70 and anti-Jo-1 were not detected. There was the presence of anti-cardiolipin at 15.3 GPL-U/ml (reference <13.3 GPL-U/ml), lupus coagulant and rheumatoid factor. Anti-liver-kidney-microsomal (LKM) antibody was not detected. Serum complement 4 was mildly depressed at 0.11 g/L (reference range 0.13–0.38 g/L), while complement 3 was normal. Direct Coomb's test was positive. The immunoglobulin profile showed hypergammaglobulinaemia at 20.8 g/L (reference internal 5.95–13.1 g/L). Urine examination did not suggest proteinuria or hematuria.

**Table 1:** A table illustrating the laboratory values of the patient.

	Result	Reference interval
Hemoglobin (g/dl)	9.6	13.4–17.1
White blood cell (×10^9^/L)	3.4	3.7–9.2
Lymphocyte (×10^9^/L)	0.8	1.0–3.1
Platelet (×10^9^/L)	202	145–370
Reticulocyte (%)	0.4	0.5–2.0
C-reactive protein (mg/L)	17	<5
Erythrocyte sedimentation ratio (mm/hr)	76	<17
Lactate dehydrogenase (IU/L)	358	118–221
Ferritin (pmol/L)	1166	32–342
Triglyceride (mmol/L)	1.3	<1.7
Albumin (g/L)	32	37–47
Globulin (g/L)	47	24–37
Aspartate transaminase (IU/L)	424	<42
Alanine aminotransferase (IU/L)	307	<51
Gamma-glutamyl transferase (IU/L)	105	<42
Alkaline phosphatase (IU/L)	272	74–290
Anti-nuclear antibodies	1:640 (speckled and cytoplasmic pattern)	
Anti-double-stranded DNA (IU/ml)	69	<50
Anti-extractable nuclear antigens		
Anti-ribonucleoprotein/Smith	Present	
Anti-Smith	Not detected	
Anti-Ro	Not detected	
Anti-La	Not detected	
Anti-Scl-70	Not detected	
Anti-Jo-1	Not detected	
Anti-cardiolipin (GPL-U/ml)	15.3	<13.3
Lupus anticoagulant	Present	
Rheumatoid factor	Present	
Complement 3 (g/L)	0.99	0.9–1.61
Complement 4 (g/L)	0.11	0.13–0.38
Direct Coomb's test	Positive	
Immunoglobulin G (g/L)	20.8	5.95–13.1

**Figure 1:**
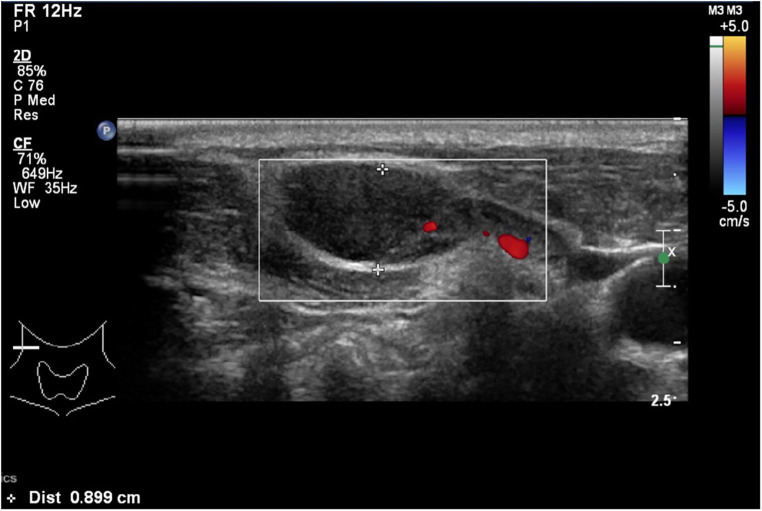
A neck ultrasound showing an enlarged lymph node which measures 1.9 cm in transverse diameter, 0.8 cm in anteroposterior diameter and 2.2 cm in crania-caudal diameter in the right upper posterior triangle region.

**Figure 2:**
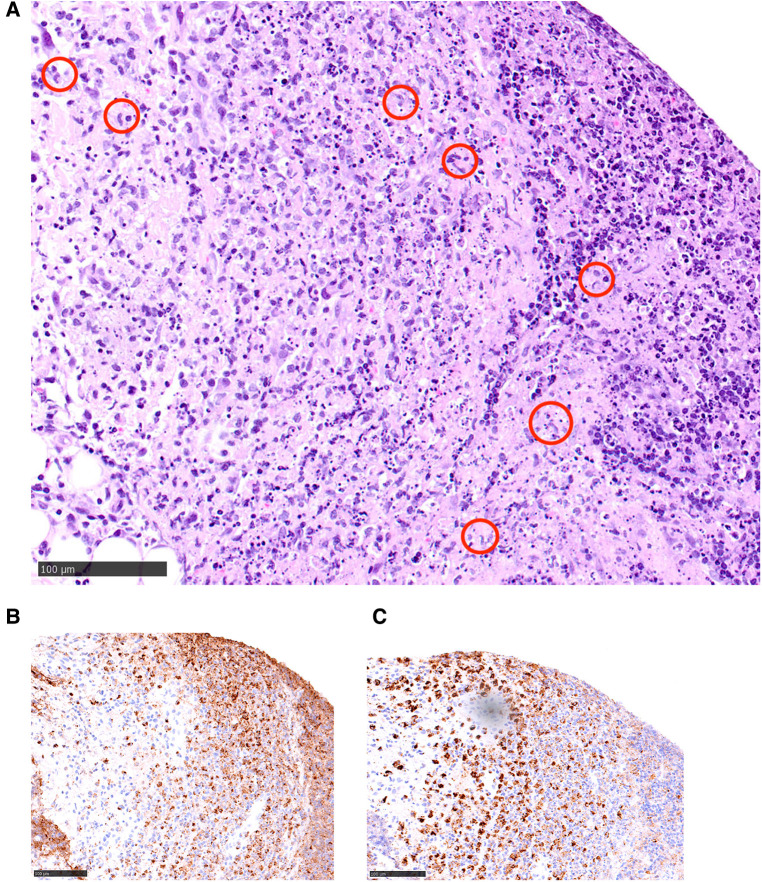
(**A**) A hematoxylin and eosin (H&E) slide illustrating a characteristic area of necrotising histiocytic lymphadenitis: there is necrosis accompanied by histiocytes (circled in red) and significant karyorrhectic debris. (**B,C**) Within the necrotic area, there are crescentic histiocytes (brown-staining cells) with dual expression of MPO (usually a marker for myeloid lineage) (**B**, left side of the picture) and PGM1 (**A** histiocyte marker) (**C**, right side) by immunohistochemistry.

His neck ultrasound revealed prominent and enlarged lymph nodes in bilateral cervical regions. The largest one measured 1.9 cm in transverse diameter, 0.8 cm in anteroposterior diameter and 2.2 cm in craniocaudal diameter in the right upper posterior triangle region (Figure 1). A lymph node biopsy was performed on day 8 of admission to rule out malignancy. The hematoxylin and eosin (H&E) slide showed necrosis accompanied by histiocytes and significant karyorrhectic debris (Figure 2A). There were crescentic histiocytes with dual expression of MPO (usually a marker for myeloid lineage) (Figure 2B) and PGM1 (a histiocyte marker) (Figure 2C) by immunohistochemistry. Neutrophils were absent. There was no evidence of malignancy. Serial sections with the Ziehl-Neelsen stain did not show acid-fast bacilli, and the Grocott stain did not show fungal organisms. The histopathological finding was suggestive of necrotising histiocytic lymphadenitis. His bone marrow aspirate and trephine biopsy did not suggest malignancy. The chest radiograph, echocardiogram and hepatobiliary ultrasound were normal.

**Figure 3:**
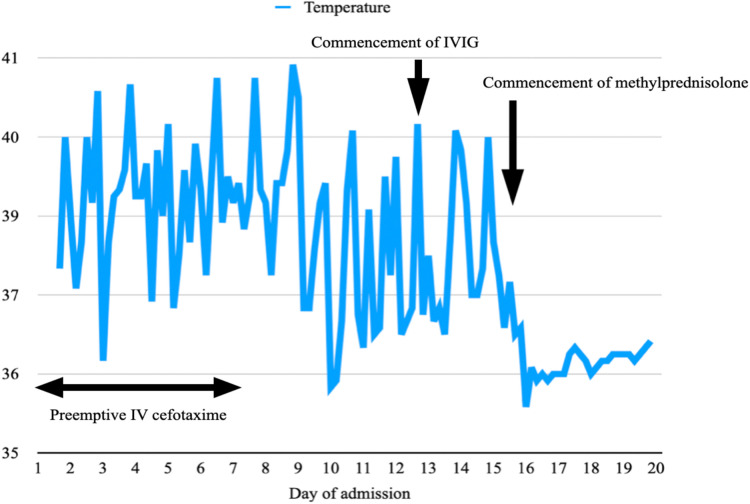
A temperature graph illustrating a remittent and intermittent fever, which persists despite a course of IV cefotaxime from day 1 to 7 of admission and IVIG on days 13 and 14. The fever settles promptly after the commencement of IV methylprednisolone on day 15 and does not recur.

**Figure 4:**
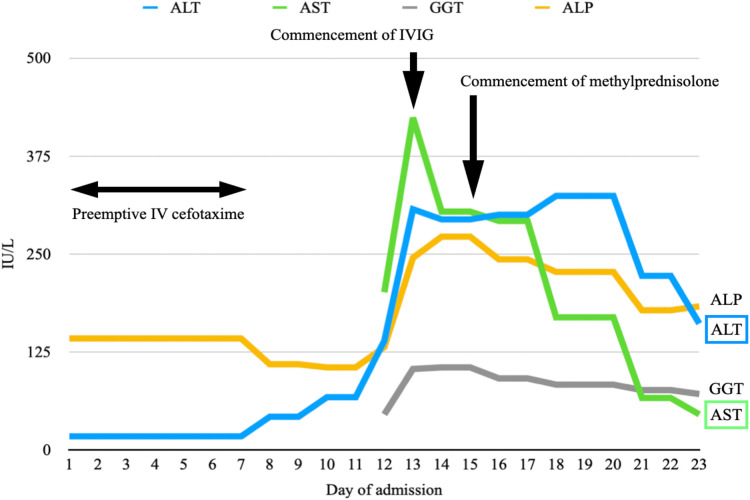
A graph illustrating the rise of predominantly ALT and AST since day 8 of admission. They show a downtrend after the commencement of IVIG on days 13 and 14 of admission. ALT elevates transiently after the commencement of methylprednisolone on day 15 before it drops again five days later. Both AST and GGT show a downtrend. ALP remains within the reference range throughout the whole of the admission.

He met three criteria of The American College of Rheumatology (ACR) 1997: hematologic disorder (leukopenia, lymphopenia), positive ANA and immunological disorders (presence anti-dsDNA, anti-cardiolipin antibodies and lupus anticoagulant).. He fulfilled the Systemic Lupus International Collaborating Clinics (SLICC) criteria, which was more sensitive especially in early childhood systemic lupus erythematosus (SLE), including leukopenia, presence of antinuclear antibodies (ANA), anti-double-stranded DNA (dsDNA), anti-cardiolipin, lupus anticoagulant, low complement 4 and positive direct Coomb's test. He was diagnosed with systemic lupus erythematosus (SLE), which manifested as necrotising histiocytic lymphadenitis and lupus hepatitis. He received preemptive intravenous (IV) cefotaxime without clinical or biochemical improvement after admission. He was treated with intravenous immunoglobulin (IVIG) of 2 grams on days 13 and 14 of admission. Liver parenchymal enzymes showed a downtrend, but his fever persisted. He was treated with intravenous methylprednisolone 50 mg every 24 h on day 15 of admission after the lymph node biopsy ruled out malignancy. The fever settled promptly after the commencement of methylprednisolone (Figure 3), and the enlarged cervical lymphadenopathy resolved in four days. ALT elevated transiently before it dropped again five days after methylprednisolone treatment (Figure 4). Both AST and GGT showed a downtrend. ALP remained within the reference range throughout the whole of the admission. The Systemic Lupus Erythematosus Disease Activity Index 2000 (SLEDAI-2K) at the time of admission was 6 which suggested an active disease, and by the time of follow up, the score decreased to 2. He had been followed for a total of 22 months so far without further recurrence. He is currently on prednisolone 2 mg daily, azathioprine 75 mg daily and hydroxychloroquine 200 mg daily to control his SLE.

## Discussion

### Multiple autoimmune syndrome (MAS)

Autoimmune disease is a systemic immune response resulting from the loss of immunological tolerance to self-antigen. It encompasses more than 100 individual diseases. They are often diagnosed according to classification criteria. However, they share common clinical manifestations, pathophysiologic mechanisms and genetic factors. This is known as an autoimmune tautology (from Greek auto, “the same” and logos, “word/idea”). The term “kaleidoscope of autoimmunity” describes the possible change of one disease to another or the fact that more than one disease may coexist in the same individual. Around 25 per cent of patients with autoimmune diseases tend to develop another autoimmune disorder (1). When more than one autoimmune disease coexists, this is defined as polyautoimmunity. When three or more autoimmune diseases coexist, this is known as multiple autoimmune syndrome (MAS).

Humbert and Dupond first described multiple autoimmune syndrome (MAS) in 1988 (2). It is classified into three groups according to the prevalence of their associations. Type I includes myasthenia gravis, thymoma, polymyositis and giant cell myocarditis. Type II groups together Sjögren's syndrome, rheumatoid arthritis, primary biliary cirrhosis, scleroderma and autoimmune thyroid disorders. Type III comprises ten autoimmune diseases, including autoimmune thyroid disease, myasthenia and, or thymoma, Sjögren's syndrome, pernicious anaemia, idiopathic thrombocytopenic purpura, Addison's disease, insulin-dependent diabetes, vitiligo, autoimmune hemolytic anaemia, systemic lupus erythematosus (SLE). Subsequent studies evaluating polyautoimmunity have found associations between other autoimmune diseases which were not reported in the original report. Our patient has three autoimmune diseases, namely type 1 diabetes mellitus, Hashimoto's thyroiditis and childhood-onset SLE, which are a rare combination among MAS type III.

The cohort of 388 patients from the Childhood Arthritis and Rheumatology Research Alliance (CARRA) Legacy Registry showed that the prevalence of polyautoimmunity in childhood-onset SLE was 8.8% (3). Another retrospective review of 1,463 Brazilian pediatric patients demonstrated a similar prevalence of 9.8%. Hashimoto thyroiditis, autoimmune hepatitis and type 1 diabetes mellitus accounted for 29%, 18% and 15.9%, respectively. Other reported autoimmune diseases in lupus patients included antiphospholipid syndrome, autoimmune vitiligo, coeliac disease, Sjogren syndrome, autoimmune gastritis, primary sclerosing cholangitis and myasthenia gravis (4). Most patients were diagnosed with SLE within one year of symptom onset. Polyautoimmunity was associated with a higher hospitalisation rate and more aggressive immunotherapy use. However, it appeared to have no impact on the SLE activity and quality of life over time.

Research has identified several risk factors for developing polyautoimmunity. First, acute and chronic forms of stress facilitate the autoimmune process by increasing inflammatory markers in the blood. Stressed individuals were around three times more likely (95% Cl, 9.2 per 1000) to develop some form of autoimmunity vs. the non-stressed cohort (95% Cl, 2.99–3.25 per 1,000), as shown by the Swedish retrospective cohort study in 2018. In addition, they are more likely to develop multiple autoimmune diseases (5). The meta-analysis by Steptoe in 2017 suggested a modest increase in circulating inflammatory markers, including interleukin (IL)-1β, IL-2, IL-8, tumour necrosis factor (TNF)-α, C-reactive protein, interferon (IFN)-γ following laboratory-induced psychological stress (6) these elevated inflammatory markers exacerbated by stress may lead to multiple autoimmune diseases. Second, viruses, typically Epstein-Barr virus and cytomegalovirus, predispose the development of autoimmunity. There are two main hypotheses, namely molecular mimicry and bystander activation. In molecular mimicry, individual T cell exhibits receptors for both foreign and self-antigens. The T cell also attacks healthy body tissues when a foreign insult triggers it. This has consistently been linked to polyautoimmunity and multiple autoimmune diseases (7). Another hypothesis that explains how viruses predispose to autoimmune diseases suggests that a virus induces a systemic and non-specific activation of the immune system. This leads to an overexcitability state and autoreactive immunopathology. This is known as bystander activation (8). Third, the PTPN22 gene is responsible for regular T-cell receptor signalling pathways. Its mutation is an important factor in MAS. Familial autoimmunity is also a factor that is significantly associated with MAS. Individuals with a family history of autoimmune disease, who are genetically susceptible to autoimmune diseases, stressed or infected with multiple viruses, have a significant risk of developing multiple autoimmune diseases. A whole genome sequencing may be considered in our patient.

### Necrotising histiocytic lymphadenitis (Kikuchi disease) and childhood-onset SLE

Childhood-onset systemic lupus erythematosus (SLE) is a chronic autoimmune disease with heterogeneous manifestations. The incidence and prevalence are 0.3–0.9 per 100,000 children-years and 3.3–8.8 per 100,000 children, respectively (9). The median age of onset is between 11 and 12 years. About 80% of patients are female (10).

Lymphadenopathy is a common finding in SLE at some point during the course of the disease, but it is rarely the primary presenting feature. In most cases, no lymph node biopsy is performed; therefore, the histopathological diagnosis remains unknown. The only study in which a biopsy of lymphadenopathy associated with SLE was performed reported that 20% of cases displayed histological findings indistinguishable from Kikuchi disease (11).

Kikuchi-Fujimoto's disease, also known as histiocytic necrotising lymphadenitis, is a benign and self-limited disease that typically presents with cervical lymphadenopathy and fever. The disease was first described in young women from Japan by Kikuchi and Fujimoto in 1972 (12). The aetiology is not clearly known. The underlying pathogenesis is proposed to be an immune response to viruses due to its self-limiting clinical course and the lack of a neutrophilic response. A recent retrospective review of 98 children by *SN Selvanathan* et al. in 2019 showed a male predominance in children with a ratio of 1.13:1, different from that seen in adults. Laboratory findings are non-specific. Mild leukopenia occurs in 30%–70% of cases (13). Antinuclear antibodies (ANA) are positive at the time of diagnosis in 30% of cases (14). A lymph node biopsy is necessary for histopathological diagnosis. It typically shows patchy necrosis of the lymph node with surrounding foamy histiocytes.

The systemic review by Bernardo Sopeña in 2017 has identified 113 patients with Kikuchi disease associated with SLE (15). The patients ranged from 14 to 56 years old and are commonly Asians. SLE was diagnosed before Kikuchi disease in 20 cases (18%), Kikuchi disease and SLE were simultaneously diagnosed in 58 cases (51%), and the onset of SLE occurred after that of Kikuchi disease in 35 cases (31%). The two diseases share a common clinical presentation. Lymphopenia is the most frequent hematological manifestation. While the diagnosis of Kikuchi's disease is histological, the diagnosis of SLE is based on a set of clinical and laboratory findings. In both conditions, a corticosteroid is commonly adopted as the cornerstone treatment. Other treatments have shown to be effective include hydroxychloroquine, intravenous immunoglobulin and rituximab in severe cases.

Our patient presents with pyrexia of unknown origin and predominantly cervical lymphadenopathy, which are common presentations of both Kikuchi disease and SLE. Despite the fact that he lacks the typical manifestations of SLE, including dermopathy, joint disease, neurological disorder and serositis, he fulfils one clinical criterion (leukopenia) and five immunological criteria (presence of ANA, anti-dsDNA, anti-cardiolipin, lupus anticoagulant, low complement 4 and positive direct Coomb's test) among the SLICC criteria for the diagnosis of SLE. The presence of hepatitis is more suggestive of SLE than Kikuchi disease. However, being the highly specific autoantibodies in SLE, anti-dsDNA is only mildly elevated at his initial presentation, and anti-Sm is absent. Instead, he has the presence of anti-RNP/Sm and rheumatoid factor, which are commonly present in mixed connective tissue disease and rheumatoid arthritis, respectively. Hence it is important to remain vigilant for an overlap syndrome with SLE, mixed connective tissue disease and rheumatoid arthritis.

## Conclusion

This patient illustrated a rare combination of multiple autoimmune syndrome comprising type 1 diabetes, Hashimoto thyroiditis and childhood-onset systemic lupus erythematosus. Pediatrician should remain vigilant for multiple autoimmune syndrome and possible overlap syndrome as autoimmune diseases commonly share similar subphenotypes and non-specific autoantibodies.

## Data Availability

'The original contributions presented in the study are included in the article/Supplementary Materials, further inquiries can be directed to the corresponding author/s.

## References

[1] Madan Mohan P Ramesh TC. Multiple autoimmune syndrome. Indian J Dermatol Venereol Leprol. (2003) 69:298–9. 17642919

[2] Humbert P Dupond JL. Multiple autoimmune syndromes. Ann Med Interne. (1988) 139(3):159–68. 10.1111/j.1365-2133.1997.tb14972.x 3059902

[3] AlAhmed O Sivaraman V Moore-Clingenpeel M Ardoin SP Bout-Tabaku S, CARRA registry investigators. Autoimmune thyroid diseases, autoimmune hepatitis, celiac disease and type 1 diabetes mellitus in pediatric systemic lupus erythematosus: results from the CARRA legacy registry. Lupus. (2020) 29(14):1926–36. 10.1177/0961203320961469 33016198

[4] Setoue DN Pitta AC Fiorot FJ Nastri MM Novak GV Molinari BC Symptomatic polyautoimmunity at diagnosis of 1463 childhood-onset lupus: a Brazilian multicenter study. Autoimmun Rev. (2018) 17(8):836–9. 10.1016/j.autrev.2018.03.009 29885968

[5] Song H Fang F Tomasson G Arnberg FK Mataix-Cols D de la Cruz LF Association of stress-related disorders with subsequent autoimmune disease. JAMA. (2018) 319(23):2388–400. 10.1001/jama.2018.7028 29922828 PMC6583688

[6] Steptoe A Hamer M Chida Y. The effects of acute psychological stress on circulating inflammatory factors in humans: a review and meta-analysis. Brain Behav Immun. (2007) 21(7):901–12. 10.1016/j.bbi.2007.03.011 17475444

[7] Cusick MF Libbey JE Fujinami RS. Molecular mimicry as a mechanism of autoimmune disease. Clin Rev Allergy Immunol. (2012) 42(1):102–11. 10.1007/s12016-011-8294-7 22095454 PMC3266166

[8] Münz C Lünemann JD Getts MT Miller SD. Antiviral immune responses: triggers of or triggered by autoimmunity? Nat Rev Immunol. (2009) 9(4):246–58. 10.1038/nri2527 19319143 PMC2854652

[9] Kamphuis S Silverman ED. Prevalence and burden of pediatric-onset systemic lupus erythematosus. Nat Rev Rheumatol. (2010) 6:538–46. 10.1038/nrrheum.2010.121 20683438

[10] Hiraki LT Benseler SM Tyrrell PN Hebert D Harvey E Silverman ED. Clinical and laboratory characteristics and long-term outcome of pediatric systemic lupus erythematosus: a longitudinal study. J Pediatr. (2008) 152(4):550–6. 10.1016/j.jpeds.2007.09.019 18346514

[11] Kojima M Motoori T Asano S Nakamura S. Histological diversity of reactive and atypical proliferative lymph node lesions in systemic lupus erythematosus patients. Pathol Res Pract. (2007) 203:423–31. 10.1016/j.prp.2007.03.002 17540509

[12] Kikuchi M. Lymphadenitis showing focal reticulum cell hyperplasia with nuclear debris and phagocytosis: a clinicopathologic study. Acta Hematol Jpn. (1972) 35:379–80.

[13] Dorfman RF Berry GJ. Kikuchi’s histocytic necrotising lymphadenitis: an analysis of 108 cases with emphasis on differential diagnosis. Semin Diagn Pathol. (1988) 5:329–45. 3217625

[14] Dumas G Prendki V Haroche J Amoura Z Cacoub P Glacier L Kikuchi–Fujimoto disease retrospective study of 91 cases and review of the literature. Medicine (Baltimore). (2014) 93:372–82. 10.1097/MD.0000000000000220 25500707 PMC4602439

[15] Sopeña B Rivera A Chamorro A Freire M Alende V Seco E Clinical association between Kikuchi’s disease and systemic lupus erythematosus: a systematic literature review. Semin Arthritis Rheum. (2017) 47(1):46–52. 10.1016/j.semarthrit.2017.01.011 28233572

